# Selective Decrease of Components of the Creatine Kinase System and ATP Synthase Complex in Chronic Chagas Disease Cardiomyopathy

**DOI:** 10.1371/journal.pntd.0001205

**Published:** 2011-06-28

**Authors:** Priscila Camillo Teixeira, Ronaldo Honorato Barros Santos, Alfredo Inácio Fiorelli, Angelina Morand Bianchi Bilate, Luiz Alberto Benvenuti, Noedir Antonio Stolf, Jorge Kalil, Edecio Cunha-Neto

**Affiliations:** 1 Laboratory of Immunology, Heart Institute, School of Medicine, University of São Paulo, São Paulo, Brazil; 2 Division of Clinical Immunology and Allergy, School of Medicine, University of São Paulo, São Paulo, Brazil; 3 Institute for Investigation in Immunology (iii), INCT, São Paulo, Brazil; 4 Division of Surgery, Heart Institute, School of Medicine, University of São Paulo, São Paulo, Brazil; 5 Division of Pathology, Heart Institute, School of Medicine, University of São Paulo, São Paulo, Brazil; René Rachou Research Center, Brazil

## Abstract

**Background:**

Chronic Chagas disease cardiomyopathy (CCC) is an inflammatory dilated cardiomyopathy with a worse prognosis than other cardiomyopathies. CCC occurs in 30 % of individuals infected with *Trypanosoma cruzi,* endemic in Latin America. Heart failure is associated with impaired energy metabolism, which may be correlated to contractile dysfunction. We thus analyzed the myocardial gene and protein expression, as well as activity, of key mitochondrial enzymes related to ATP production, in myocardial samples of end-stage CCC, idiopathic dilated (IDC) and ischemic (IC) cardiomyopathies.

**Methodology/Principal Findings:**

Myocardium homogenates from CCC (N = 5), IC (N = 5) and IDC (N = 5) patients, as well as from heart donors (N = 5) were analyzed for protein and mRNA expression of mitochondrial creatine kinase (CKMit) and muscular creatine kinase (CKM) and ATP synthase subunits aplha and beta by immunoblotting and by real-time RT-PCR. Total myocardial CK activity was also assessed. Protein levels of CKM and CK activity were reduced in all three cardiomyopathy groups. However, total CK activity, as well as ATP synthase alpha chain protein levels, were significantly lower in CCC samples than IC and IDC samples. CCC myocardium displayed selective reduction of protein levels and activity of enzymes crucial for maintaining cytoplasmic ATP levels.

**Conclusions/Significance:**

The selective impairment of the CK system may be associated to the loss of inotropic reserve observed in CCC. Reduction of ATP synthase alpha levels is consistent with a decrease in myocardial ATP generation through oxidative phosphorylation. Together, these results suggest that the energetic deficit is more intense in the myocardium of CCC patients than in the other tested dilated cardiomyopathies.

## Author Summary

Chronic Chagas disease cardiomyopathy (CCC) affects millions in endemic areas and is presenting in growing numbers in the USA and European countries due to migration currents. Clinical progression, length of survival and overall prognosis are significantly worse in CCC patients when compared to patients with dilated cardiomyopathy of non-inflammatory etiology. Impairment of energy metabolism seems to play a role in heart failure due to cardiomyopathies. Herein, we have analyzed energy metabolism enzymes in myocardium samples of CCC patients comparing to other non-inflammatory cardiomyopathies. We found that myocardial tissue from CCC patients displays a significant reduction of both myocardial protein levels of ATP synthase alpha and creatine kinase enzyme activity, in comparison to control heart samples, as well as idiopathic dilated cardiomyopathy and ischemic cardiomyopathy. Our results suggest that CCC myocardium displays a selective energetic deficit, which may play a role in the reduced heart function observed in such patients.

## Introduction

Chagas disease is a significant cause of morbidity and mortality in Central and South America, affecting about 13 million people [1]. The disease is caused by infection with the intracellular protozoan parasite *Trypanosoma cruzi*. About 30 % of infected patients develop chronic Chagas disease cardiomyopathy (CCC), an inflammatory cardiomyopathy that occurs decades after the initial infection. One-third of CCC patients further progress to a particularly aggressive, life-threatening dilated cardiomyopathy; CCC is a major indication of heart failure in Latin America [2], [3]. Clinical progression, length of survival and overall prognosis are significantly worse in CCC patients when compared to patients with dilated cardiomyopathy of non-inflammatory etiology, like idiopathic dilated or ischemic cardiomyopathies (IDC or IC, respectively) [4]–[7]. Due to migration from endemic countries, an estimated 300,000 people with Chagas disease are living in the USA, where a significant number of cases of CCC are expected per year [8]. The pathogenesis of CCC is unclear, and multiple mechanisms have been proposed (Reviewed in [9]). The most characteristic histopathological lesions in cardiac patients with CCC are consistent with inflammation and a myocardial remodeling process: T cell/macrophage-rich myocarditis, hypertrophy, and fibrosis with cardiomyocyte damage [10], [11]. The local cytokine production profile shows a T1-type response, with interferon-gamma-induced chemokines [12]–[15]. As the currently licensed anti-*T. cruzi* drugs may not be effective in preventing the progression of heart lesions of CCC [16], treatment is only supportive. In patients with refractory heart failure, the only available treatment is heart transplantation [17]. The absence of alternative treatment for CCC is a consequence of limited knowledge about the pathogenesis.

Energy metabolism imbalances have been reported in dilated cardiomyopathies and heart failure [18]. Since the heart consumes more energy than any other organ, impairments in energy production could lead to a mechanical failure of the heart, and disturbances in electrical conduction [18]. Mitochondrial oxidative phosphorylation is essential for the production of energy for cardiac function. This system comprises the oxidative phosphorylation complex, which includes the electron transport chain (complexes I to IV) and the F_1_F_O_ ATP synthase (complex V). In aerobic tissues, most ATP is synthesized via the mitochondrial F_1_F_O_ ATP synthase complex. Studies have shown that certain components of oxidative phosphorylation may be impaired in heart failure [18]. Patients with IDC or IC show a reduced myocardial activity of complex III when compared to controls [19]. In IDC patients, a decreased activity of myocardial cytochrome c oxidase (complex IV) was observed [20]. With the progression of dilated cardiomyopathy, higher levels of spatial and functional heterogeneity within mitochondrial populations are observed, indicative of mitochondrial damage [21]. It has been reported that mitochondrial damage leads to loss of mitochondrial function, impairing energy production and cell physiology, and to the enhancement of pathologic function, producing oxidative-, calcium-, apoptosis-mediated myocyte injury [22].

Creatine kinases (CK) are also key enzymes of energy metabolism, which connect mitochondrial ATP-producing and cytosolic ATP-consuming process, and are thus of central importance to the cellular energy homeostasis [23]. This system acts as an energy buffer, in which mitochondrial creatine kinase (CKMit) catalyzes the transfer of high energy phosphate bond from ATP to creatine to form phosphocreatine and ADP. Phosphocreatine, a molecule smaller than ATP, diffuses rapidly from the mitochondria to the myofibrils, where myofibrillar creatine kinase (MM, MB, BB dimers, formed by CKM, the muscular isoform, and CKB, the brain isoform) catalyzes ATP production from phosphocreatine, generating free creatine, which diffuses back into mitochondria [18], [23]. Impaired ATP transfer and utilization may limit contractile function by means of a decrease in the average cytoplasmic ATP concentration [18]. Most of the components of the CK system are down-regulated in heart failure, with levels of creatine, phosphocreatine, CKMit and CKM all significantly reduced in animal models and in humans [24], [25]. CK deficiency in isolated hearts may cause a decline of over 70 % in ATP delivery to myofibrils, leading to a blunted contractile reserve [26].

Proteomic profiling of myocardium from CCC patients revealed that 27 % of identified proteins belong to energy metabolism pathways [27]. Using gene expression profiling, our group found differential expression of a significant number of genes involved in oxidative phosphorylation and lipid catabolism in myocardial samples from CCC patients, but not in samples from patients with dilated cardiomyopathy, when compared to samples from subjects without cardiomyopathy [15]. Genetic profiling studies showed that hearts of *T. cruzi*-infected mice have shown a decreased expression of oxidative phosphorylation enzymes [28]. Likewise, biochemical and histochemical analysis revealed a reduced activity of the respiratory chain complexes in hearts of *T. cruzi*-infected mice [29]. Proteomic analysis of myocardial samples from acutely *T. cruzi*-infected Syrian hamsters showed an up-regulation of the energy metabolism proteins glutamate oxaloacetate transaminase 1 and pyruvate dehydrogenase β, that may be associated with a high ATP demand after *T. cruzi* infection [30].

Inflammatory cytokines, which are present in the CCC myocardium and induce local signaling [12]–[15], have been reported to alter the energy metabolism. IFN-gamma was shown to inhibit the mitochondrial oxidative metabolism [31] and increase the rate of cardiac ATP depletion in cardiomyocytes [32]. Additionally, studies with cultured human skeletal muscle cells demonstrated that IFN-gamma treatment could inhibit CK activity [33].

Thus, the evidence is consistent with the hypothesis that the myocardium of patients with CCC could present an impaired energy metabolism. In order to test this hypothesis, we compared the protein and mRNA expression of CKM, CKMit, and the alpha and beta subunits of the catalytic F_1_ domain of ATP synthase complex (ATPα and ATPβ, respectively) in myocardial samples from CCC, with that of dilated cardiomyopathies of other etiologies, and healthy hearts from organ donors. We also measured total creatine kinase enzymatic activity in the same sample groups.

## Methods

### Samples of human myocardium

Myocardial samples were obtained from left ventricular-free wall heart tissue from end-stage heart failure patients at the moment of heart transplantation. Samples from 5 CCC (at least 2 positive results in 3 independent anti-*T. cruzi* serology tests –ELISA immunoassay, indirect immunofluorescence assay and indirect hemagglutination test), 5 IDC (dilated cardiomyopathy in the absence of ischemic disease, negative serology for Chagas disease) and 5 coronary angiography-proven IC patients were collected (Table 1). Left ventricular free wall samples were also obtained from healthy hearts of organ donors, which were not used for transplantation for technical reasons. The protocol was approved by the Institutional Review Board of the University of São Paulo School of Medicine and written informed consent was obtained from the patients. Samples were cleared from pericardium and fat, quickly frozen in liquid nitrogen and stored at −70°C. Protein homogenates were obtained using lysing solution (1∶10 w/v) containing 7 mol/L urea, 10 mmol/L Tris, 5 mmol/L magnesium acetate and 4 % CHAPS, pH 8.0, by mechanical homogenization (PowerGen, Fisher Scientific). For experiments measuring the creatine kinase enzyme activity, 20 mg of tissue was lysed in solution (1∶20 w/v) containing 0.32 mol/L sucrose, 10 mmol/L HEPES and 1 mmol/L EDTA, pH 7.4, by mechanical homogenization. The homogenate was then sonicated for three cycles of 10 s each to 10 Watts (60 Dismembrator Sonic, Fisher Scientific), centrifuged at 12,000 g for 30 min. Supernatants were collected and stored at −70°C. Protein quantification was performed with the Bradford method (BioRad).

**Table 1:** Baseline characteristics of included patients.

Etiol.*	Patient	Sex	AGE	EF†	LVDD‡	Fibrosis§	Myocarditis||
N	#1	M	40	nd	nd	0	0
N	#2	M	22	nd	nd	0	0
N	#3	M	46	nd	nd	0	0
N	#4	M	17	nd	nd	0	0
N	#5	M	28	nd	nd	0	0
CCC	#6	M	50	11	82	2+	2/3+
CCC	#7	M	49	37	77	3+	3+
CCC	#8	M	28	21	68	2+	2/3+
CCC	#9	M	58	29	64	2+	2+
CCC	#10	M	57	29	71	1+	2/3+
IDC	#11	M	38	16	88	0/1+	0
IDC	#12	M	53	19	77	1/2+	0
IDC	#13	M	55	25	51	3+	0
IDC	#14	M	56	16	99	2+	0
IDC	#15	M	36	14	62	0/1+	0
IC	#16	M	62	37	75	2/3+	0
IC	#17	M	61	33	79	3+	0
IC	#18	M	52	20	62	3+	0
IC	#19	M	63	25	74	2+	0
IC	#20	M	49	25	76	1+	0

*Etiol.: Etiology.

†EF: Ejection Fraction (reference value: ≥55%).

‡LVDD: Left Ventricular Diastolic Diameter (reference value: 39–53 mm).

§and || as rated by histopatology (0: absent, 1 +: mild, 2 +: moderate, 3 +: intense).

N: individuals without cardiomyopathies.

CCC: chronic Chagas disease cardiomyopathy.

IDC: idiopathic dilated cardiomyopathy.

IC: ischemic cardiomyopathy.

M: Male.

nd: not detected.

### Myocardial histopathology

The samples from myocardial tissue (left ventricular free wall) were fixed in buffered formalin solution (pH 7.2), embedded in paraffin, and cut into 5 µm sections. Sections were stained with hematoxylin-eosin (H&E) and picrosirius red.

### Analysis of protein expression by Immunoblotting

Extracts of myocardial samples containing 30 µg of protein were heated for 5 min at 95°C, and subjected to one-dimensional electrophoresis (SDS-PAGE) using 12.5 % polyacrylamide gel and the vertical electrophoresis system Ruby SE600 (GE Healthcare). After electrophoresis, proteins were transferred from gel to a nitrocellulose membrane using the TE Semi-Dry Transfer Unit (GE Healthcare). The nitrocellulose membranes were incubated with monoclonal antibodies to proteins involved in energy metabolism: anti- F_1_F_O_ ATP synthase alpha (ATPα) and anti- F_1_F_O_ ATP synthase beta (ATPβ) (Molecular Probes), anti-mitochondrial creatine kinase (CKMit) and anti- muscle creatine kinase (CKM) (Santa Cruz) and polyclonal anti- glyceraldehyde 3-phosphate dehydrogenase (GAPDH) (R & D Systems). Each membrane was subjected to incubation with compatible secondary antibodies conjugated with peroxidase, developed using ECL Plus Western Blotting Detection Reagents (GE Healthcare) and detection using X-ray equipment. Analysis of densitometry was performed using the program ImageQuant TL (GE Healthcare).

### Analysis of mRNA expression by real-time reverse transcriptase (RT)-PCR

Total RNA from left ventricle samples was isolated using the RNeasy Fibrous tissue kit (Quiagen). Contaminating DNA was removed by treatment with RNase-free DNase I. cDNA was obtained from 5 µg total RNA using Super-script II™ reverse transcriptase (Invitrogen). mRNA expression was analyzed by real-time quantitative reverse transcriptase (RT)-PCR with SYBR Green I PCR Master Mix (Applied Biosystems) and 250 nM of sense and anti-sense primers using the ABI Prism 7500 Real Time PCR System (Applied Biosystems). The following primers were designed using Primer Express software version 3.0 (Applied Biosystems): GAPDH (M33197): (F) 5′-TGGTCTCCTCTGACTTCAACA-3′, (R) 5′-AGCCAAATTCGTTGTCATACC-3′; ATPα (NM_001001937): (F) 5′-TCTTCAAAAGACTGGGACTGCTGA-3′, (R) 5′-AAGACACGCCCAGTTTCTTCAAG-3′; ATPβ (NM_001686): (F) 5′-GCCCAGCATTTGGGTGAGA-3′, (R) 5′-GATTGGTGCACCAGAATCCAGT-3′; CKM (NM_001824): (F) 5′-GCTCTCTGTGGAAGCTCTCAACA-3′, (R) 5′-GATGAGCTGCTGCTGCTCCT-3′; CKMit (NM_001825): (F) 5′-TGACGAGGAGTCCTATGAGGTGTT-3′, (R) 5′-AGATCCGTTGTGTGCTTCATCAC-3′. After every PCR, an amplicon melting point curve was obtained. This yielded a single peak with the expected temperature provided by Primer Express software, confirming the specificity of the PCR. GAPDH mRNA expression was used for normalization. The amount of mRNA in the left ventricles samples was calculated using the 2^-ΔCt^ method [34].

### Measurement of the creatine kinase enzymatic activity

The enzymatic activity measurements of CK in the myocardial samples were performed using the CK-NAC kit (Doles). Basically, this method is a kinetic system where CK catalyzes the transphosphorylation reaction of ADP to ATP. A series of coupled enzymatic reactions produce NADH in a concentration directly proportional to the enzymatic activity of CK in the sample. The analyses were performed using a UV/Vis U-2001 spectrophotometer (Hitachi), monitoring the increase in absorbance of NADH per minute at the wavelength of 340 nm at 37°C, using a thermostatic bath (MultiTemp III, GE Healthcare). The measurement of enzymatic activity is given in international units (U); one unit of CK is the amount of enzyme that oxidizes 1 µmol/L of NADH per minute. Values were normalized by the amount of protein present in the sample.

### Statistical analysis

All statistical analyses were performed with GraphPad Prism 4.0 software (GraphPad Software). Descriptive statistics are given as average and standard deviation. The non-parametric Newman-Keuls test was used for comparison between the groups. P-values <0.05 were considered as statistically significant.

## Results

**Figure 1:**
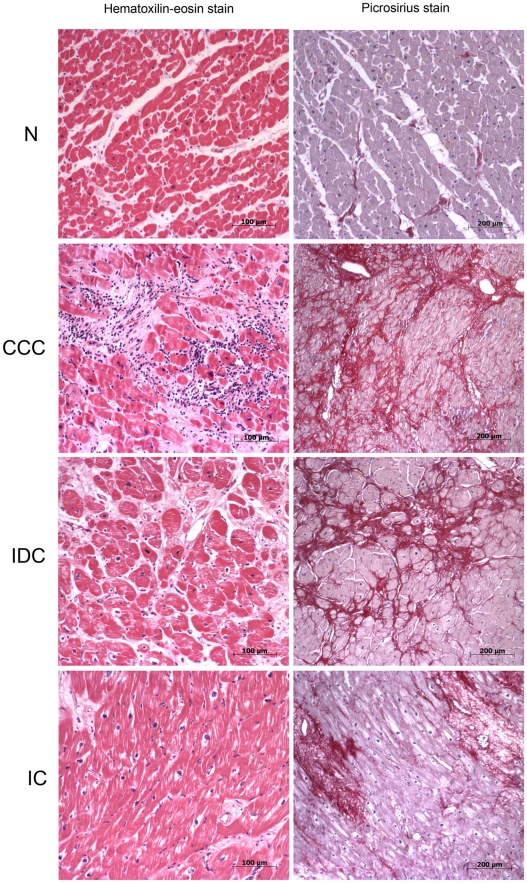
Histopathological features of myocardial samples. Slides of hematoxilin-eosin- (left column) and picrosirius red- (right column) stained myocardial sections of representative patients with CCC, IDC and IC and individuals without cardiomyopathies (N). Myocardial hypertrophy characterized by fiber and nuclear enlargement is evident in the CCC, IDC and IC groups. Lymphocytic myocarditis is present only in the CCC group. Interstitial fibrosis stained red in picrosirius stain is present in the CCC, IDC and IC groups.

While myocardial sections from all 3 cardiomyopathy groups displayed cardiomyocyte hypertrophy and fibrosis upon histopatholological analysis, myocarditis associated with a predominant lymphocytic infiltration was only observed among CCC heart lesions (Figure 1, Table 1). No significant differences were found in age, ejection fraction (EF) or left ventricular diastolic diameter (LVDD) among the three cardiomyopathy groups.

**Figure 2:**
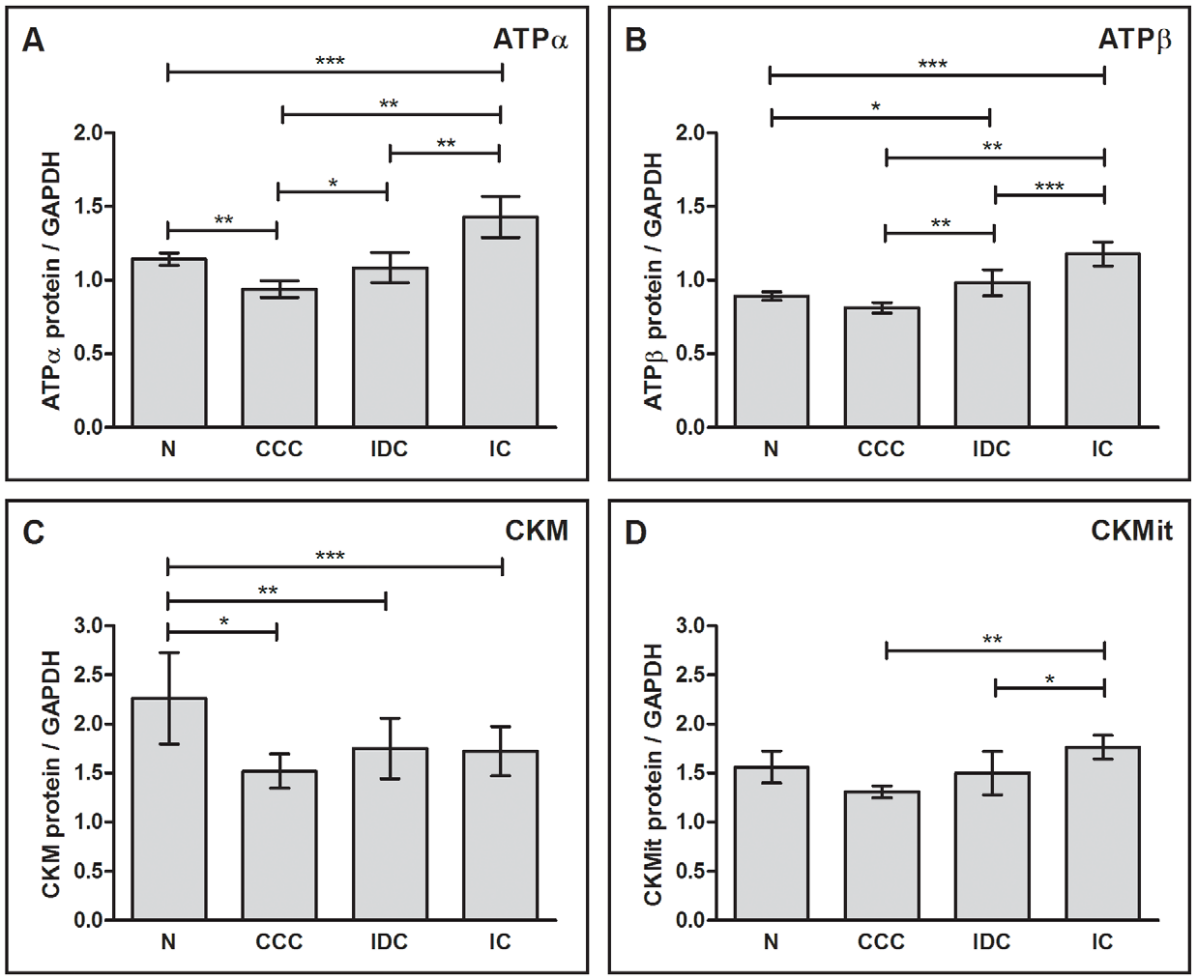
Analysis of differential protein expression of energy metabolism enzymes by immunoblotting. (A) ATPα: ATP synthase alpha subunit, (B) ATPβ: ATP synthase beta subunit, (C) CKM: creatine kinase M and (D) CKMit: mitochondrial creatine kinase. The densitometric values of each protein for each sample were normalized by the values of GAPDH. The horizontal lines show statistically significant changes: *p<0.05; **p<0.01; ***p<0.001.

Figures 2 A and B show the differential protein expression of the ATP-synthase, subunits alpha (ATPα) and beta (ATPβ), respectively. Representative immunoblots are depicted in Figure S1. The ATPα was 18 % less expressed in CCC myocardium when compared to myocardial samples from individuals without cardiomyopathies (p<0.01). In contrast, IC myocardium showed an increase of 25 % (p<0.001) in ATPα when compared to control samples, while in IDC there was no significant reduction of ATPα levels (5 %, p = ns) in comparison to the control group. In the comparison between cardiomyopathy groups, ATPα levels in CCC were 34 % lower than in IC myocardium (p<0.01); and 13 % lower than those found in IDC myocardium (p<0.05). There was no significant decrease of ATPβ in CCC when compared to control samples (9 %, p = ns). However, we observed increased expression of ATPβ in IC and IDC myocardium when compared to the control group [32 % (p<0.001) and 10 % (p<0.05), respectively]. In the comparison between cardiomyopathy groups, we found that CCC myocardium samples express significantly less ATPβ than IC or IDC myocardium [31 % (p<0.001) and 17 % (p<0.01), respectively].

We have also detected significant protein expression differences among enzymes of the creatine kinase system. The expression of CKM (Figure 2C) was decreased in samples from patients with CCC (33 %, p<0.01), IDC (23 %, p<0.05) and IC (24 %, p<0.05) when compared to the control group. Of note, in the comparison between cardiomyopathy groups, the average CKM expression was most decreased among CCC patients (13 % and 12 % reduction when compared to IDC and IC, respectively), although the difference was not statistically significant. The protein expression of CKMit (Figure 2D) was decreased in samples from patients with CCC and IDC, when compared to the control group (16 % and 4 %, respectively), but the differences failed to achieve statistical significance. In contrast, samples from patients with IC showed an increased expression of CKMit when compared to the control group (13 %; p = ns). In the comparison between cardiomyopathy groups, the protein levels of CKMit were decreased in samples from patients with CCC when compared to samples from patients with IC and IDC [26 % (p<0.01) and 13 % (p = ns), respectively].

**Figure 3:**
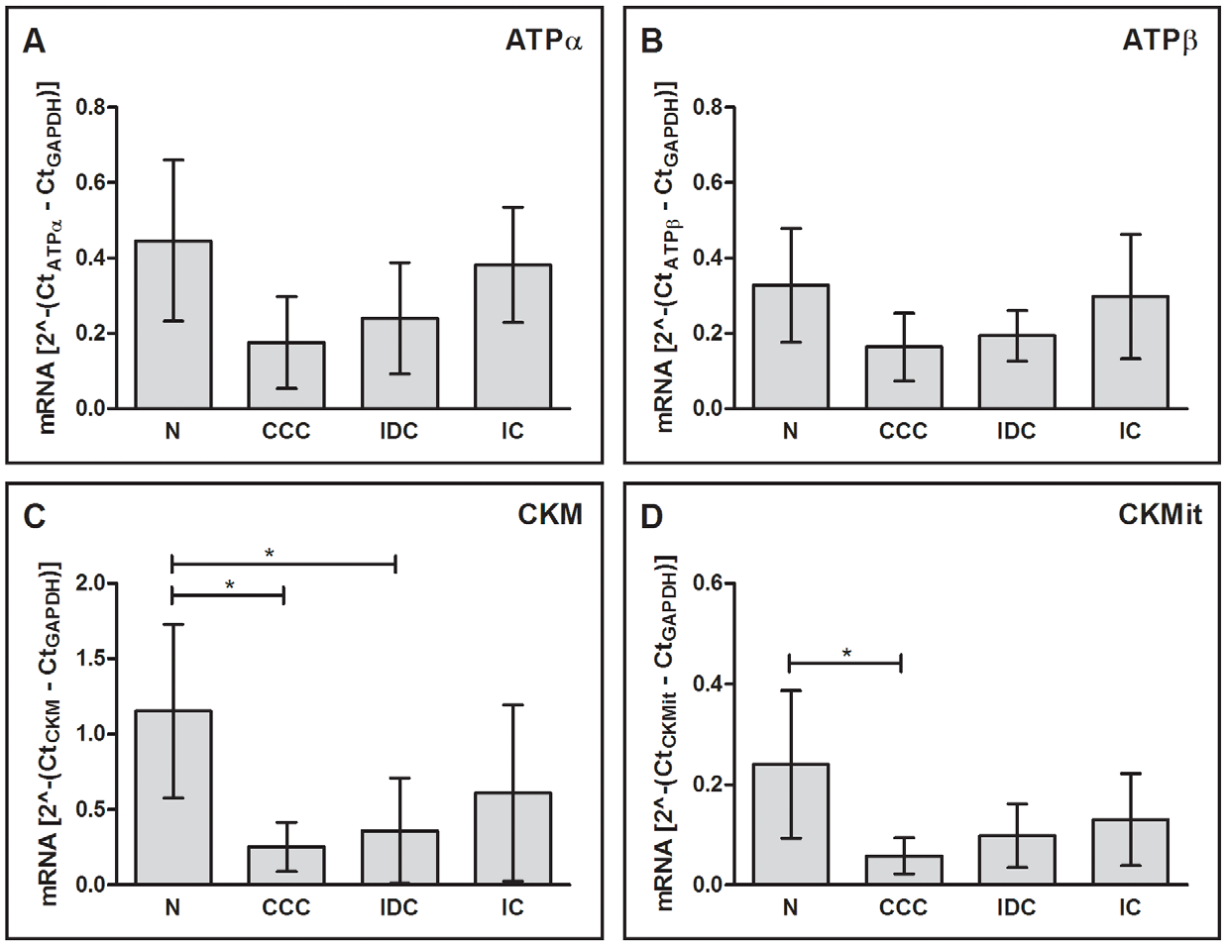
mRNA expression of energy metabolism enzymes by real time RT-PCR. (A) ATPα: ATP synthase alpha subunit, (B) ATPβ: ATP synthase beta subunit, (C) CKM: creatine kinase M and (D) CKMit: mitochondrial creatine kinase. The horizontal lines show statistically significant changes: *p<0.05.

We also analyzed the mRNA expression of the enzymes tested above, ATPα, ATPβ, CKM and CKMit. Figure 3 shows the values of relative quantification of mRNA expression of these enzymes. We found that mRNA expression of CKM was decreased in samples from patients with CCC and IDC when compared to samples from subjects without cardiomyopathy [78 % (p<0.05) and 69 % (p<0.05)]. Also, the mRNA expression of CKMit was reduced in CCC samples (75 %, p<0.05) when compared to samples from subjects without cardiomyopathy. The average expression of mRNAs for all 4 enzymes was reduced in the 3 cardiomyopathies when compared to control samples, and samples from CCC patients showed the lowest expression levels. However, due to high interindividual variation of expression within each group, most of the comparisons were not statistically significant. Interestingly, mRNA levels of ATPα and ATPβ were not increased in samples from patients with IC in comparison to samples from control group, as seen in the analysis of protein expression. In the comparison between cardiomyopathy groups, none of the enzymes analyzed showed significant differences in mRNA expression for ATPα and ATPβ.

**Figure 4:**
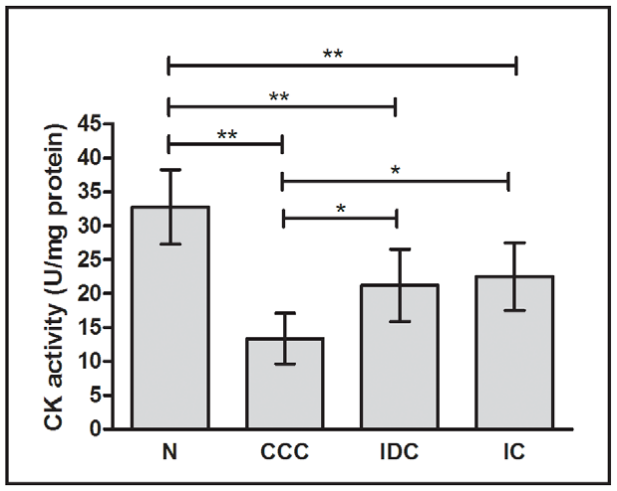
Analysis of creatine kinase enzymatic activity. The values were normalized by the amount of protein from each sample. The horizontal lines show statistically significant changes: *p<0.05; **p<0.01.

In order to evaluate whether the differential protein expression of enzymes of the creatine kinase system observed above had an impact on myocardial enzyme activity, we compared total creatine kinase activity among groups (Figure 4). The creatine kinase enzyme activity was reduced in samples from patients with CCC (59 %, p<0.01), IDC (35 %, p<0.01) and IC (31 %, p<0.01) when compared to samples from the control group (Figure 4). Of interest, creatine kinase enzyme activity was significantly reduced in myocardial samples from patients with CCC, when compared to IDC and IC patients [37 % (p<0.05) and 41 % (p<0.05), respectively].

## Discussion

In this paper, we observed a reduced expression of CKM, a key enzyme in myocardial energetic metabolism, in several dilated cardiomyopathies. Most importantly, we found that CCC myocardium shows significantly reduced levels of protein expression of ATP synthase alpha subunit and total creatine kinase enzyme activity, when compared to IDC or IC.

We observed that the myocardial creatine kinase system shows impaired function in patients with all forms of cardiomyopathy. The reduced myocardial protein expression of CKM, observed in all cardiomyopathy groups, was reflected in the reduced total creatine kinase activity. This has been previously described for IDC and IC [35]. The reduced protein expression of CKM was probably due to transcriptional regulation at least in the cases of CCC and IDC, since CKM mRNA expression was also significantly reduced in samples from patients of such groups, when compared to samples from individuals without cardiomyopathy. Animals genetically deficient in CKM develop myocardial hypertrophy and left ventricular dilation [36], as well as higher susceptibility to mitochondrial damage and cardiac disturbances in calcium homeostasis after ischemia and reperfusion [37]. Myocardial ATP flux through the CK system was shown to be reduced by 50 % in patients with heart failure [38]. Since the CK reaction is the prime source of the myocardial energy reserve, the deficit in ATP flux through CK may contribute to the pathogenesis of heart failure [39]. The finding that average CKM and CKMit levels from CCC samples were the lowest among all groups, and that total CK activity in CCC samples was significantly lower than that of the other cardiomyopathy groups, indicates that CCC patients may show a stronger functional impairment in the CK system than other etiologies of dilated cardiomyopathy. It is likely that the significantly reduced myocardial expression of CKMit, observed in comparison to IC, - and to a lesser degree also in comparison to control group - may have contributed to the reduction in the total CK activity observed in CCC samples. Regarding the discrepancy between the significantly reduced CKMit mRNA levels and the less prominent reduction of CKMit protein levels observed in CCC, it could be due to an increased stability of this protein. The loss of CK activity in isolated hearts has been reported to cause a decline in ATP delivery to myofibrils, leading to a blunted contractile reserve [26]. Reduced energy reserve via creatine kinase, as indicated by reduced phosphocreatine/ATP ratios, limits cardiac performance during metabolic stress conditions [40]. Significantly, CCC patients have been reported to display an impaired myocardial contractile response to dobutamine [41]. It is thus possible that this reduced contractile reserve is a consequence of the significant derangement in CK activity reported here in CCC myocardium.

A correlation has been reported between decreased total CK activity and LV dysfunction [42]. However, samples from CCC patients studied here showed lower total CK activity than those of IDC or IC patients, despite the fact that LV dysfunction status was similar in CCC, IC and IDC patients. This may indicate that there are disease-specific factors that induce a stronger reduction in CK activity in CCC, when compared to non-inflammatory cardiomyopathies. While the creatine kinase system may buffer transient changes in ATP levels, the rate of oxidative ATP synthesis must be closely matched to the rate of consumption. Most myocardial ATP is generated through the mitochondrial oxidative phosphorylation (complex I-V). In our study, we also found changes in the protein expression of ATPα and ATPβ (belonging to the F_1_ subunit of ATP synthase complex - complex V). The finding that ATPα was only reduced in CCC myocardium, but not in IC and IDC, indicates that these patients could be at a greater impairment in cardiac ATP supply, as suggested by studies in animal models of heart failure [43]. However, in our study, patients with IC showed elevated protein levels of ATPα and ATPβ, and patients with IDC showed elevated levels of ATPβ when compared to the myocardium of subjects without cardiomyopathy. Significantly, a study showed an increase in mRNA of oxidative phosphorylation components in chronic ischemia due to severe atherosclerosis [44].

*In vivo* measurements in normal hearts subjected to adrenergic stress have shown that ATP production by oxidative phosphorylation increases with the demand, while ATP production by the CK system remains unchanged [39]. Authors thus suggest that the ratio of ATP production by the CK system to oxidative phosphorylation decreases upon demand; in addition, the ratio may be even lower in resting hearts from heart failure patients, due to a decreased CK flux [39]. Since a reduced CK flux may be one of the most prominent metabolic abnormalities in heart failure [39], the findings of selectively reduced CK activity - and perhaps also oxidative phosphorylation activity - may suggest that energy production in CCC myocardium can be especially restricted in situations of increased demand.

Inflammation associated to the significant lymphocytic infiltrate may play an important role in multiple steps of CCC pathogenesis. Inflammatory cytokines such as IFN-gamma and TNF-alpha, abundantly produced in the inflammatory milieu of CCC heart tissue, are known to induce gene expression changes in cardiomyocytes [12], [45], [46], and may directly influence energy metabolism. It has been shown that *in vitro* treatment with IFN-gamma inhibited the oxidative metabolism [31], and increased the rate of ATP depletion in cardiomyocytes [32]. Additionally, studies with cultured human skeletal muscle cells demonstrated that IFN-gamma treatment could inhibit the CK activity [33].

In summary, we reported that CK activity and ATPα levels are significantly reduced in CCC myocardium when compared to IDC and IC samples. If confirmed by studies with a higher number of samples, one could hypothesize that these findings could contribute to the contractile dysfunction, loss of inotropic reserve and worse outcome of CCC when compared to cardiomyopathies of non-inflammatory etiology. *In vivo* analysis of CK flux rate and ATP synthesis though oxidative phosphorylation may allow further validation of the present findings.

## Supporting Information

Figure S1 **Representative immunoblotting of the proteins.** ATPα: ATP synthase alpha; ATPβ: ATP synthase beta; CKMit: mitochondrial creatine kinase; CKM: creatine kinase M; GAPDH: glyceraldehyde-3-phosphate dehydrogenase, used for normalization.
(TIF)
