# Supplementary figures and images for: Selective Decrease of Components of the Creatine Kinase System and ATP Synthase Complex in Chronic Chagas Disease Cardiomyopathy
Source: PLoS Negl Trop Dis. 2011 Jun 28;5(6):e1205. doi: 10.1371/journal.pntd.0001205 (PMC3125151; doi:10.1371/journal.pntd.0001205)

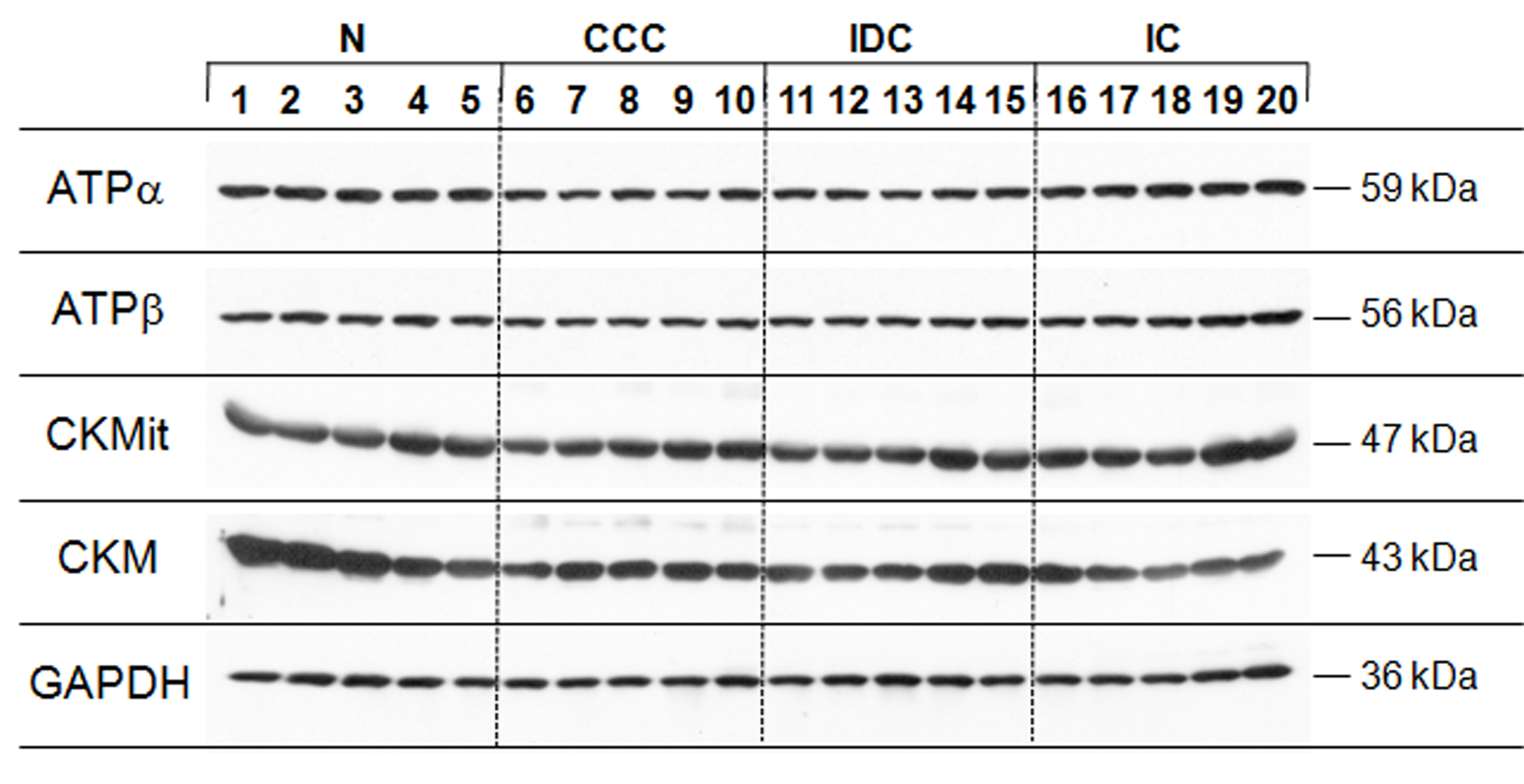

Supplement: Figure S1 — Representative immunoblotting of the proteins. ATPα: ATP synthase alpha; ATPβ: ATP synthase beta; CKMit: mitochondrial creatine kinase; CKM: creatine kinase M; GAPDH: glyceraldehyde-3-phosphate dehydrogenase, used for normalization. (TIF) [file pntd.0001205.s001.tif]
